# Diabetic foot disease carries an intrinsic high risk of mortality and other severe outcomes in type 2 diabetes: a propensity score-matched retrospective population-based study

**DOI:** 10.1186/s12933-024-02303-1

**Published:** 2024-06-19

**Authors:** Bogdan Vlacho, Magdalena Bundó, Judit Llussà, Jordi Real, Manel Mata-Cases, Xavier Cos, Diana Tundidor, Francesco Zaccardi, Kamlesh Khunti, Edward B. Jude, Josep Franch-Nadal, Dídac Mauricio

**Affiliations:** 1 DAP-Cat Group, Unitat de Suport a la Recerca Barcelona Ciutat, Institut Universitari d’Investigació en Atenció Primària Jordi Gol (IDIAP Jordi Gol), Carrer Sardenya 375, Entresuelo, 08025 Barcelona, Spain; 2 CIBER of Diabetes and Associated Metabolic Diseases (CIBERDEM), Instituto de Salud Carlos III (ISCIII), Barcelona, Spain; 3 Institut de Recerca Hospital de la Santa Creu i Sant Pau, Sant Quintí, 89, 08041 Barcelona, Spain; 4 Primary Health Care Center Ronda Prim, Gerència d’Àmbit d’Atenció Primària Metropolitana Nord de Barcelona, Institut Català de la Salut, Mataró, Spain; 5 Primary Health Care Centre Sant Roc, Gerència d’Àmbit d’Atenció Primària Metropolitana Nord de Barcelona, Institut Català de la Salut, Mataró, Spain; 6 Primary Health Care Center La Mina, Gerència d’Àmbit d’Atenció Primària Barcelona Ciutat, Institut Català de la Salut, Sant Adrià de Besòs, Spain; 7 Primary Health Care Center Sant Martí de Provençals, Gerència d’Àmbit d’Atenció Primària Barcelona Ciutat, Institut Català de la Salut, Barcelona, Spain; 8 Innovation Office, Institut Català de la Salut, Barcelona, Spain; 9 Department of Endocrinology and Nutrition, Hospital de la Santa Creu i Sant Pau, Barcelona, Spain; 10 Leicester Diabetes Research Centre, University Hospital Leicester, Leicester General Hospital, Gwendolen Rd, Leicester, LE5 4PW UK; 11 Leicester Real World Evidence Unit, Diabetes Research Centre, University of Leicester, Gwendolen Rd, Leicester, LE5 4PW UK; 12 Tameside and Glossop Integrated Care NHS Foundation Trust, Tameside on Lyne, UK; 13 University of Manchester, Manchester, UK; 14 Primary Health Care Center Raval Sud, Gerència d’Àmbit d’Atenció Primària Barcelona Ciutat, Institut Català de la Salut, Barcelona, Spain; 15 Department of Medicine, University of Vic - Central University of Catalonia, Vic, Spain

**Keywords:** Amputation, Cardiovascular events, Diabetic foot disease, Primary healthcare, Incidence, Severe outcomes

## Abstract

**Background:**

To evaluate the association between diabetic foot disease (DFD) and the incidence of fatal and non-fatal events in individuals with type 2 diabetes (T2DM) from primary-care settings.

**Methods:**

We built a cohort of people with a first DFD episode during 2010–2015, followed up until 2018. These subjects were 1 to 1 propensity score matched to subjects with T2DM without DFD. The incidence of all-cause mortality, the occurrence of new DFD, amputations, cardiovascular diseases, or composite outcome, including all-cause mortality and/or cardiovascular events during the follow-up period, were calculated. A Cox proportional hazard analysis was conducted to evaluate the hazard ratios (HR) for different events.

**Results:**

Overall, 11,117 subjects with T2DM with a first episode of DFD were compared with subjects without DFD. We observed higher incidence rates (IRs) for composite outcome (33.9 vs. 14.5 IR per 100 person-years) and a new DFD episode event (22.2 vs. 1.1 IR per 100 person-years) in the DFD group. Compared to those without DFD, those with a first episode of DFD had a higher HR for all events, with excess rates particularly for amputation and new DFD occurrence (HR: 19.4, 95% CI: 16.7–22.6, HR: 15.1, 95% CI: 13.8–16.5, respectively) was found.

**Conclusions:**

Although DFD often coexists with other risk factors, it carries an intrinsic high risk of morbidity and mortality in individuals with T2DM. DFD should be regarded as a severe complication already at its onset, as it carries a poor clinical prognosis.

**Graphical Abstract:**

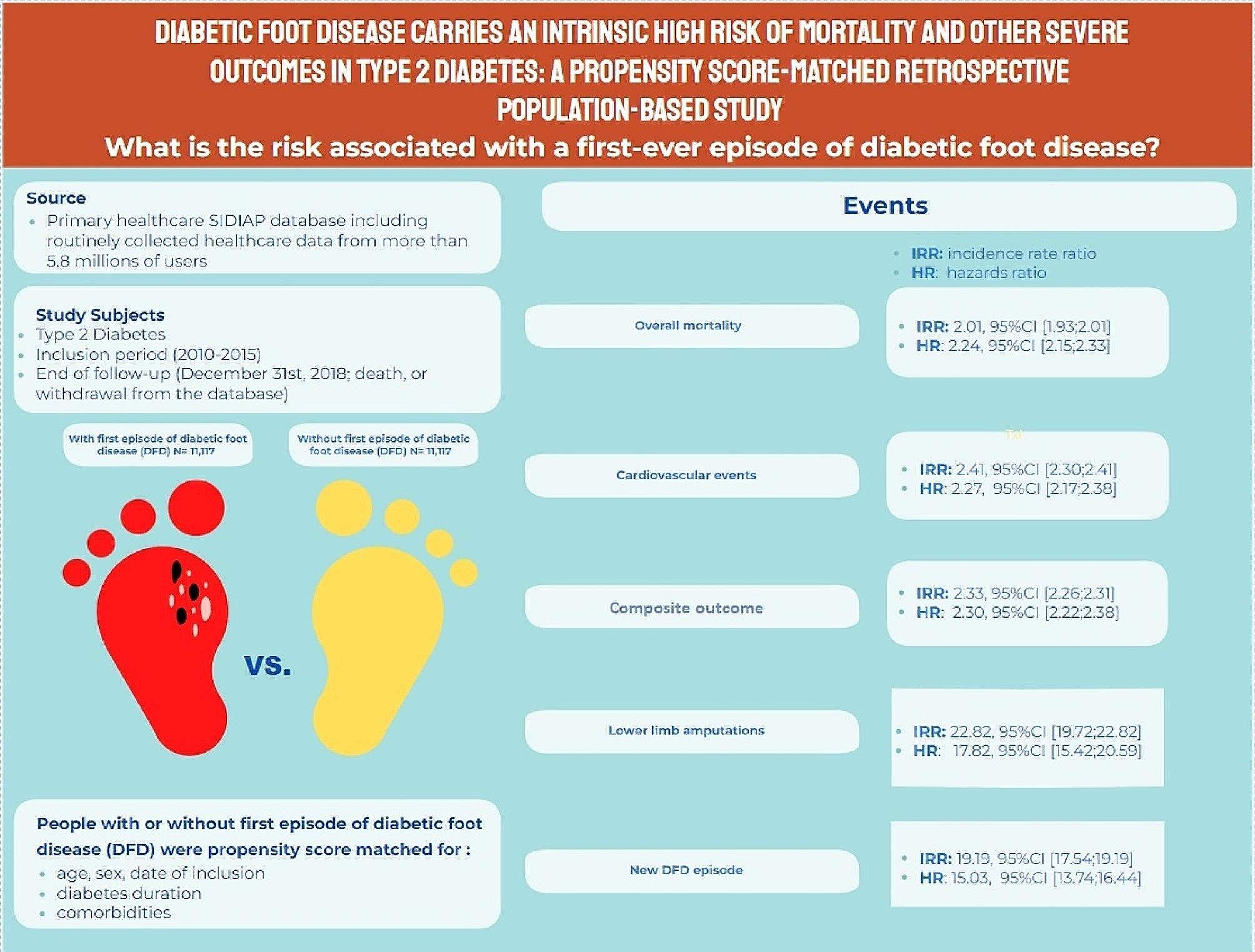

**Supplementary Information:**

The online version contains supplementary material available at 10.1186/s12933-024-02303-1.

## Background

Type 2 diabetes mellitus (T2DM) is one of the most prevalent chronic diseases worldwide, associated with decreased quality of life, reduced life expectancy, and higher healthcare costs [1]. Diabetic foot disease (DFD) is a serious complication of T2DM, imposing a substantial burden on patients and healthcare expenses [2]. Furthermore, this condition confers a higher risk of mortality than other common diseases, such as breast or colon cancer [3].

Diabetic foot ulcer (DFU) is one of the most common forms of DFD and increases the risk of infection and amputation [2]. It is estimated that 19–35% of people with T2DM will develop a DFU during their lifetime, with 50–60% becoming infected and 20% eventually requiring amputation [4]. Around 10% of these individuals will die during the first year after an initial DFU, and 5 years survival rates are only 50–60% [4, 5]. While there have been reports of a decline in the incidence of DFU and a reduction in the number of amputations, especially major amputations, likely due to improved care for people with diabetes [6], there remains significant variability in the incidence of amputations and mortality as a result of DFD among different countries or even within the same country [7].

Several studies have reported that DFD is a significant risk factor for major clinical outcomes such as cardiovascular disease, lower-extremity amputation, and mortality [8–12]. However, many important variables increase the risk of complications, making its prevention and management difficult. Many clinical factors have been determined to be associated with a poor prognosis in patients with a DFD, including age, duration of diabetes, glycaemic control, cardiovascular risk factors, and micro- and macrovascular complications [4]. In the absence of properly designed studies, it remains unclear what the impact of each of those factors is on the patient’s prognosis, and whether DFD could be a prognostic factor independent of other risk factors of a serious underlying pathology [4]. So far, one retrospective cohort study [13] using a matching technique to control for baseline characteristics (age and sex) in subjects with DFU and diabetes (both type 1 and type 2) with control patients with diabetes who had no previous history of foot ulcer, osteomyelitis, or amputation found a higher morbidity, mortality, and excess healthcare costs among the people with DFU was reported [13]. However, the patients were only matched on age and sex, and other risk factors were not considered.

The availability of healthcare databases in recent years has provided investigators with valuable information, due to the comprehensive clinical information from a large number of subjects, which allow for insights into health conditions and their evolution over time. Previously, we reported that risk factors such as male sex, duration of diabetes, diabetes complications and previous history of DFD were associated with the presence of DFD in our primary care population [14]. Further to that study, we undertook the current study with the aim of investigating the association between DFD and the incidence of fatal and non-fatal outcomes in a primary-care database of individuals with T2DM in Catalonia (Spain). The analysis included a large cohort of patients with T2DM with and without DFD matched for important prognostic factors.

## Methods

### Study design and settings

We conducted a longitudinal retrospective cohort study, following the RECORD guidelines (checklist included in the **supplement material**) in individuals with a first-ever episode of DFD compared to individuals with T2DM without this condition. The primary healthcare SIDIAP database was used: SIDIAP (*Sistema d’Informació per al desenvolupament de la Investigació en AtencióPrimària*) is a well-validated database widely used in epidemiological and pharmaco-epidemiologic research on diabetes in Spain [15, 16]. It contains routinely collected healthcare data from electronic medical records of individuals attending primary healthcare centres under the *Institut Català de la Salut* (ICS). ICS is the major primary healthcare provider in Catalonia, covering over 80% of the population in the region. The inclusion period for the cohort was defined from January 1st, 2010, to December 31st, 2015.

### Study subjects

We selected all subjects from the database with a diagnosis of T2DM during the inclusion period. T2DM was defined by the presence of ICD-10 diagnostic codes (International Statistical Classification of Diseases and Related Health Problems, 10th Revision) [17] E11 and E14. Subjects with other types of diabetes (ICD-10: type 1 diabetes, E10; malnutrition-related diabetes, E12; gestational diabetes, O24; other specific types of diabetes, E13) were excluded from the analysis. During the inclusion period (2010–2015), we selected two groups of study subjects: a group of individuals with a first episode of DFD, and a group of individuals without this condition. People with DFD were identified using the presence of diagnostic codes and sub-codes for lower-extremity ulcers (L97, E11.621), osteomyelitis (M86), gangrene (I96, E11.52), lower-extremity amputation (Z89), surgical detachment procedures (0Y6), or Charcot neuroarthropathy (M14.6, E11.61): this method for identifying people with DFD was previously published [14] (Supplementary Table 1.1).

### Variables and study events

At the index date, we collected information on different variables, including age, sex, and lifestyle data (current tobacco and alcohol consumption). In addition, clinical variables were collected in the period of one year before the index date (including blood pressure, body mass index (BMI), laboratory parameters, presence of comorbidities, and concomitant medication). The presence of hypertension and hyperlipidaemia were identified based on diagnostic codes and/or prescribed treatments for each condition. Cardiovascular disease and diabetic complications (micro and macrovascular) were identified by their corresponding diagnostic codes. Chronic kidney disease was defined by the recorded diagnostic codes and/or a CKD-EPI glomerular filtration rate < 60 ml/min/1.73m^2^ and/or an albumin/creatinine ratio > 30 mg. Definitions of these clinical variables and health conditions were previously published [14].

During the follow-up period from the index date until of end of follow-up (December 31st, 2018; death, or withdrawal from the database), we collected data on different outcomes. As a single outcome, we collected data on new episodes of DFD or reoccurrence of DFD (ICD-10 diagnostic codes for lower-extremity ulcers and/or osteomyelitis and/or gangrene and/or lower-extremity amputation and/or surgical detachment procedures related to lower-extremity amputations and/or Charcot neuroarthropathy), cardiovascular disease (ICD-10 diagnostic codes for stroke, ischemic heart disease, peripheral artery disease, heart failure), amputations (ICD-10 diagnostic codes for lower-extremity amputation and/or surgical detachment procedures related to lower-extremity amputations) and all-cause mortality. We also analysed the composite outcome defined by the combination of cardiovascular disease and/or all-cause mortality. Cardiovascular disease includes peripheral artery disease as an additional adverse outcome and other cardiovascular diseases, a more comprehensive clinically pertinent outcome for the subjects with diabetes [18]. The codes used to define these events are summarized in Supplementary Table 1.2.

### Statistical methods

We applied a propensity score matching (PSM) technique to account for the differences in prognostic factors between the two groups (with and without DFD): predefined baseline variables included age, sex, inclusion year (date), diabetes duration, and the presence of the following comorbidities: hypertension, hyperlipidaemia, microvascular complications (retinopathy, neuropathy, chronic kidney disease) and macrovascular complications (peripheral artery disease, ischemic heart disease, stroke, heart failure, ). The MatchIt R package [19] was used with a nearest neighbour matching method applied to all variables except inclusion year (exact method). The approach included sampling without replacement, distance-based generalized linear model with logit link, no calliper, and a 1:1 ratio. We estimated the standardized differences of variables included in the propensity score, and produced density and PP-plots for the distribution of probability points in our PSM.

Categorical variables were reported as numbers and frequencies and continuous variables as mean and standard deviation. We calculated the number of events, person-years, incidence rates (IRs), and incidence rate ratios (IRRs) (with 95% confidence intervals (CIs)) comparing the two groups of individuals with and without DFD. We performed Cox proportional hazard regression models to estimate hazard ratios (HRs) (unadjusted and adjusted) for different events. The adjusted HRs considered variables such as age at index date, sex, smoking status, alcohol consumption, diabetes duration, hypertension, hyperlipidaemia, chronic renal disease, retinopathy, microvascular complications, and macrovascular complications, categorized BMI and HbA1c levels (< 8%/64 mmol/mol, ≥ 8%/64 mmol/mol). Missing data were addressed as following: (1) for dichotomous variables and events, such as health problems or drugs, a lack of information in the database was considered as an absence of those conditions or drugs and no missing were presented; (2) for laboratory parameters and BMI, the lack of values was considered as missing (presented as “missing” in the tables; (3) for the PSM we did not consider the missing categories due to the risk of losing potential study subjects; (4) we addressed variables with potential missing data, such as smoking status, HbA1c, and BMI, by introducing a ‘missing’ category in the adjustment of the models. Additionally, we performed a sub-analysis according to the severity of DFD comparing people with foot ulcer only with those subjects with more severe forms of DFD. Subjects with diabetic foot ulcers included only those with a first episode of foot ulcer. The group with more severe forms of DFD included those with a first episode of conditions such as osteomyelitis, gangrene, Charcot neuroarthropathy, lower-extremity amputation and surgical detachment procedures related to lower-extremity amputations. We used different statistical analysis and matching R statistical software packages [20–22]. All analyses were conducted using R statistical software version 4.3 (R Core Team 2022).

## Results

The flowchart of the study is shown in Supplement Fig. 1. A total of 389,944 individuals who met the study criteria were identified during the inclusion period. Among them, 11,117 individuals experienced a first-ever episode of DFD and were matched to those without DFD. Supplement Fig. 2.1 and 2.2 shows the characteristics of the matching variables before and after the PSM.

### Characteristics of the subjects after matching

After matching for age, sex, inclusion year, diabetes duration, and the presence of comorbidities, the clinical characteristics of the subjects were comparable between the two groups (Table 1**)**. In the overall population, more than half of the subjects (56.7%) at the moment of inclusion were at least 75 years old, majority males (57.4%) and had a diabetes duration on average of 10.1years (± 7.37). Well balanced groups were created in terms of baseline clinical characteristics and relevant risk factors for the DFD. People with DFD were younger, were more frequently current smokers, had higher BMI, exhibited poorer glycaemic control and a less favourable lipid profile, and had lower glomerular filtration rates compared to people without a first episode of DFD.

**Table 1:** Baseline characteristics of the study participants at index date

	Total	Without DFD	With DFD	*p*-values
**Age, sex, and lifestyle**	*N* = 22,234	*N* = 11,117	*N* = 11,117	
Age, years, mean (± SD) *	75.2 (11.4)	75.6 (10.9)	74.9 (11.8)	< 0.001
Age ≥ 75 years, n (%)	12,616 (56.7)	6404 (57.6)	6212 (55.9)	0.051
Sex, male, n (%) *	12,759 (57.4)	6369 (57.3)	6390 (57.5)	0.216
Current smoker, n (%)	2668 (12.0)	1195 (10.7)	1473 (13.2)	< 0.001
Smoking status missing, n (%)	1145 (5.15)	494 (4.44)	651 (5.86)	
Current alcohol intake, at risk, n (%)	3461 (15.6)	1810 (16.3)	1651 (14.9)	< 0.001
Alcohol intake missing, n (%)	9620 (43.3)	4852 (43.6)	4768 (42.9)	
**Physical examination, mean (SD)**				
BMI, kg/m^2^	30.0 (5.59)	29.8 (5.04)	30.2 (6.15)	< 0.001
Systolic blood pressure, mmHg	134 (17.5)	135 (16.4)	134 (18.5)	0.057
Diastolic blood pressure, mmHg	71.8 (10.6)	72.0 (10.2)	71.7 (10.9)	0.008
**Comorbidities**				
Diabetes duration at index date, years, mean (SD)*	10.1 (7.37)	10.1 (7.42)	10.1 (7.32)	0.594
Hypertension, n (%) *	17,897 (80.5)	9185 (82.6)	8712 (78.4)	< 0.001
Hyperlipidemia, n (%) *	14,394 (64.7)	7163 (64.4)	7231 (65.0)	0.944
Ischemic heart disease, n (%) *	5065 (22.8)	2551 (22.9)	2514 (22.6)	0.565
Heart failure, n (%) *	4607 (20.7)	2303 (20.7)	2304 (20.7)	1.000
Stroke, n (%) *	3988 (17.9)	2019 (18.2)	1969 (17.7)	0.392
Peripheral artery disease, n (%) *	7018 (31.6)	3439 (30.9)	3579 (32.2)	0.045
Macrovascular complications, n (%)	11,753 (52.9)	5767 (51.9)	5986 (53.8)	0.003
Diabetic neuropathy, n (%) *	2631 (11.8)	1283 (11.5)	1348 (12.1)	0.184
Diabetic retinopathy, n (%) *	4478 (20.1)	2220 (20.0)	2258 (20.3)	0.536
Chronic kidney disease, n (%) *	10,801 (48.6)	5491 (49.4)	5310 (47.8)	0.016
Microvascular complications, n (%)	10,128 (45.6)	5007 (45.0)	5121 (46.1)	0.128
**Laboratory parameters**				
HbA1c, (%), mean, (SD) HbA1c, mmol/mol, (SD)	7.42 (1.56) 57.6 (12.1)	7.22 (1.36) 55.4 (10.4)	7.64 (1.73) 60.0 (13.5)	< 0.001
HbA1c ≥ 8%, n (%)	4351 (28.6)	1839 (23.2)	2512 (34.6)	< 0.001
Total cholesterol (mg/dL), mean (SD)	176 (41.7)	177 (38.8)	175 (44.7)	< 0.001
HDL cholesterol (mg/dL), mean (SD)	47.4 (13.6)	48.1 (13.3)	46.7 (13.9)	< 0.001
LDL cholesterol (mg/dL), mean (SD)	100 (33.1)	101 (31.7)	99.8 (34.7)	0.210
Triglycerides (mg/dL), mean (SD)	149 (96.1)	148 (93.3)	150 (99.1)	0.314
eGFR, mL/min/1.73m^2^, mean (SD)	62.7 (21.7)	63.9 (20.5)	61.4 (22.7)	< 0.001

BMI: body mass index; DFD: diabetic foot disease; eGFR: estimated glomerular filtration rate; HbA1c: glycosylate hemoglobin; HDL: high-density lipoprotein; LDL: Low-density lipoprotein n: number; SD: standard deviation

*Variables used for propensity score matching

### Study events

The average follow-up duration in our cohort was of 3.78 (± 2.31) years. Higher incidence rates (IRs) of almost all study events were observed during the follow-up period among individuals with DFD compared to those without a first episode of DFD. These IRs were especially high for composite cardiovascular outcome (33.9 vs. 14.5 IR per 100 person-year, respectively) and a new DFD episode (22.2 vs. 1.1 IR per 100 person-year, respectively). Comparing the incidence rate ratios (IRRs) for different events, the IRR was especially high for amputation events among people with a first episode of DFD: this ratio was 23 times higher (IRR: 22.8, 95% CI: 19.7; 22.8) compared to those without DFD. Table 2 shows the study events during the follow-up period.

**Table 2:** Incidence of different study events

	New DFD episode		Amputations		All-cause mortality		Cardiovascular disease		Composite cardiovascular outcome	
	Without DFD	With DFD	Without DFD	With DFD	Without DFD	With DFD	Without DFD	With DFD	Without DFD	With DFD
Number of events	544	4906	200	2669	3998	6113	3052	4495	5862	8364
Person years	46913.65	22051.29	47412.95	27722.38	47711.56	36340.73	40334.09	24649.71	40334.09	24649.71
IR [95% CI] *	1.16 [1.06;1.26]	22.25 [21.63;22.88]	0.42 [0.37; 0.48]	9.63 [9.27;10.00]	8.38 [8.11;8.64]	16.82 [16.40;17.25]	7.57 [7.30;7.84]	18.24 [17.71;18.78]	14.53 [14.16;14.91]	33.93 [33.21;34.67]
IRR [95% CI]	19.19 [17.54;19.19]		22.82 [19.72;22.82]		2.01 [1.93;2.01]		2.41 [2.30;2.41]		2.33 [2.26;2.33]	

CI: confidential interval; DFD: diabetic foot disease; IR: incidence rate; IRR: incidence rate ratio; Composite cardiovascular outcome: all-cause mortality and/or cardiovascular disease

*Per 100 person/years

**Fig. 1:**
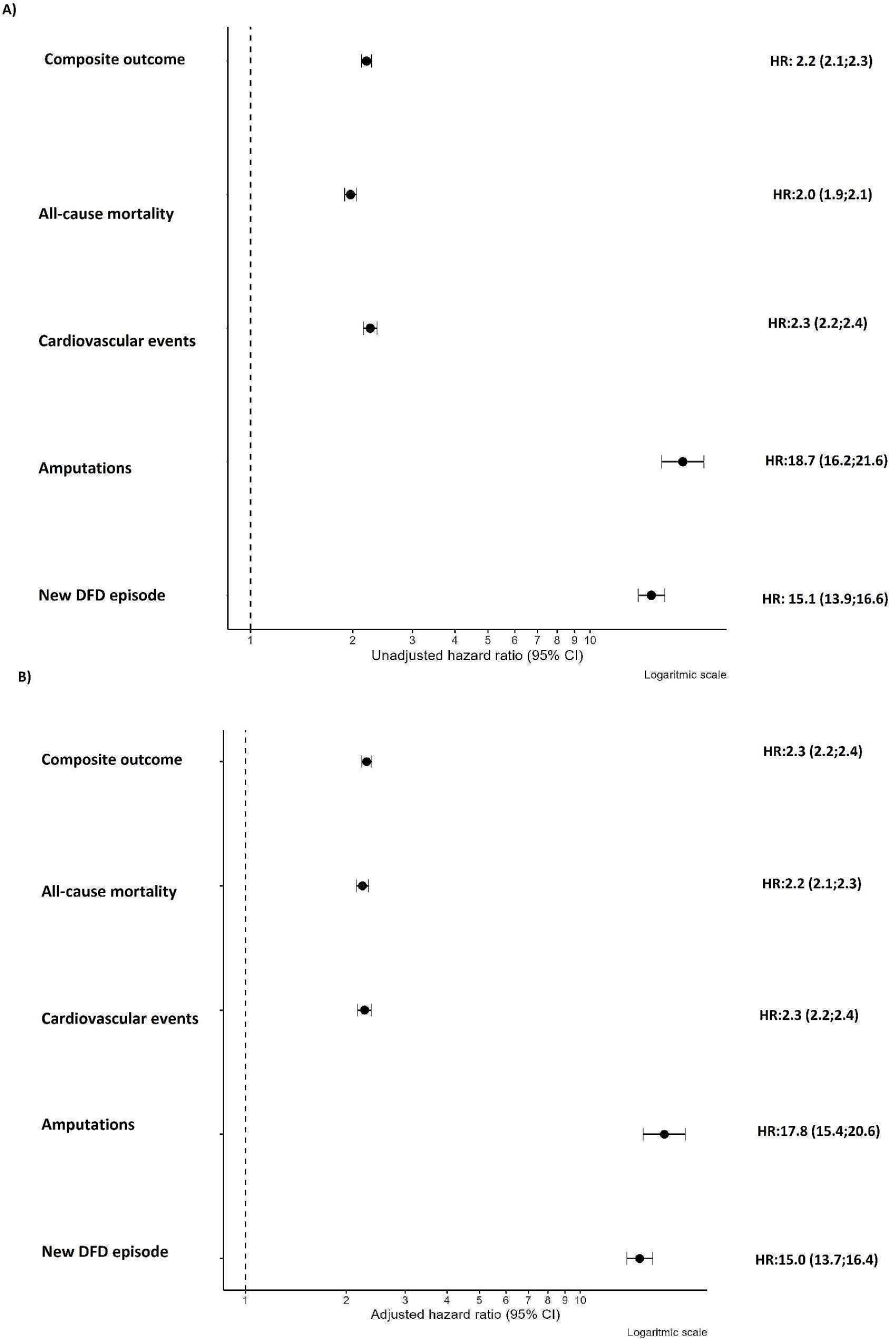
Unadjusted and adjusted hazard ratios for different study events.  **A** Unadjusted hazards ratios for different clinical outcomes **B** Adjusted hazards ratios for different clinical outcomes

In the proportional hazards analysis, individuals with DFD had higher rates for all events. The risk was particularly increased for amputation (HR: 18.74, 95% CI: 16.22; 21.64) and the occurrence of a new DFD episode (HR: 15.14, 95% CI: 13.85; 16.56). Even after adjusting for different risk factors, some events still showed an increased risk, including cardiovascular events (HR: 2.27, 95% CI: 2.17; 2.38), composite outcome (HR: 2.30, 95% CI: 2.22; 2.38), and overall mortality (HR: 2.24, 95% CI: 2.15; 2.33). Figure 1 and Supplement Table 2 summarize the adjusted and unadjusted hazard ratios (HRs).

### Sub-analysis of study events according to the severity of the initial diabetic foot disease condition

In the sub-analysis stratified by the severity of diabetic foot disease (DFD), those individuals with more severe forms of DFD (osteomyelitis, gangrene, Charcot neuroarthropathy, or lower-extremity amputation) had a higher incidence of new DFD episodes, amputations, and cardiovascular events compared with those with less severity of DFD as a first episode (just a foot ulcer). After adjusting for different risk factors, only the risk of amputations and a new DFD episode remained higher among those with more severe forms of DFD (Supplementary Table 3).

## Discussion

In our analysis, after accounting for potential concomitant risk factors, we found a notably higher risk of non-fatal events (including cardiovascular diseases, and amputations) among people experiencing a first episode of DFD. Similarly, an increased risk was observed for fatal events (such as major adverse cardiovascular events and all-cause mortality) when compared to matched individuals without DFD.

In the current study, the incident rate of a new DFD episode during the follow-up period in the DFD group was 22.3 per 100 person-years. People with previous diabetic foot ulcer are known to have a high recurrence rate of the next ulcer episode, reaching 40% within the first year and up to 65% in five years [23]. In the Eurodiale study [24], the incidence of DFD recurrence among people with diabetes was 57.5% at three years. The reason for such a high recurrence rate of DFD could be due to a number of factors including inadequate treatment of the first episode [23]. In a previous study done by our group [14], we found that the main risk factor for this DFD was a previous history of this condition (odds ratio 13.9; 95%CI: 11.81–14.72).

Regarding the amputations, it is estimated that 80% of amputations are preceded by DFD [25]. The incidence of amputations among patients with diabetes is highly variable from one country to another, and even within the same country [7, 9]. In our analysis, the incidence rate ratio for amputation events among people with a first episode of DFD was 22.8 times higher compared to those without this condition. It is well-established that having a foot ulcer is a significant risk factor for lower extremity amputation [7, 26, 27]. A study conducted by Margolis et al. [28] using US Medicare data of diabetic patients, reported that approximately 5% per year of those with a foot ulcer required an amputation. The percentage of amputations increased up to 13% in patients with osteomyelitis, while the incidence of lower extremity amputations in the subpopulation of people with diabetes and peripheral artery disease was approximately four times higher compared to the overall diabetes population. In our real-world primary care study, due to the nature of the records, the presence of foot infection at the time of the first episode of DFD was difficult to estimate, and peripheral artery disease is likely under-reported due to the lack of precise and standardized diagnostic criteria in primary care [29]. Recently published results by our group, in the study to evaluate the incidence of diabetic foot ulcers in Catalonia, we found that 64.5% of people had peripheral artery disease in cases where it was properly explored/diagnosed by pulse palpation and ankle-brachial index [30].

It has previously been reported that people with DFD have a higher mortality risk [10, 13, 31]. In the current study, during the follow-up period, the mortality incidence rate among those with a first episode of DFD was double compared to those without DFD (16.8% vs. 8.2%, respectively). This finding aligns with previous studies. Boyko et al. [32], in a prospective study with 725 diabetic subjects followed for an average of 1.8 years, observed a relative risk (RR) of death of 2.39 (95% CI 1.13 to 4.58) in subjects who developed a foot ulcer compared to those who did not. Walsh et al. [33], in a study involving 414,523 people with diabetes over 5 years, found a fully adjusted HR of 2.48 (95% CI: 2.43, 2.54) after controlling for major known complications of diabetes that might influence mortality. The meta-analysis by Saluja et al. [11] concluded that having diabetic foot ulcer was associated with an increased risk of all-cause mortality (pooled relative risk 2.45, 95% CI 1.85–2.85).

Regarding cardiovascular events, we found a higher incidence rate among people with DFD, including the composite outcome compared to subjects without DFD. The risk of a cardiovascular event and mortality among people with DFD was more than double to those without DFD. The higher prevalence of macrovascular disease among patients with DFD has already been previously described [12, 27, 34, 35]. In general, there is a lack of studies evaluating the incidence and risk of macrovascular complications during the follow-up among people with DFD and T2DM. Alonso et al. [36], using a database from Spain, observed an annual incidence of acute myocardial infarction of 0.4% among people with T2DM and DFU studied between 2007 and 2011. People with DFU in that study had a much lower prevalence of comorbidities than the current study.

To our knowledge, none of the previously mentioned studies used a matching approach to properly evaluate the intrinsic clinical impact of DFD as a strong independent prognostic factor. However, due to the observational nature of these studies, confounding variables should be considered and controlled. One retrospective study by Ramsey et al. [13] with 8,905 people with T2DM has used this methodology, although subjects with a DFU were matched only for age and sex, and not for other clinically relevant variables such as comorbidities and diabetes duration. The authors reported a 28% mortality rate at 3 years of follow-up compared with 13% in the group without DFD.

So far, the evidence from previously published studies on DFD indicates that this complex condition is a risk factor for poor clinical prognosis [4, 36]. However, important methodological limitations are present in those studies. The utilization of matching techniques enabled us to properly consider primary baseline risk factors associated with poor outcomes in patients with DFD. The findings from our study indicate that even when controlling for important clinical baseline risk factors among people with T2DM, the first episode of DFD is a strong indicator of severe vascular disease and overall poor health, where its presence is strongly associated with severe poor prognosis. The results emphasize the importance of recognizing this factor when planning the treatment and follow-up care for these people. Additionally, an important characteristic of our study is that subjects were matched for important baseline well-known risk factors. Our findings showed that a first-ever episode of DFD was intrinsically associated to poorer outcomes beyond the classical contributing factors, such as microvascular and macrovascular complications. This leads to the concept that diabetic microangiopathy may affect also non-classical microvascular beds, e.g. any organ/tissue in the lower legs [37]. Moreover, hyperglycaemia translates not only in micro- and macrovascular damage, but also into direct cellular damage in every tissue exposed to hyperglycaemia potentially leading to cellular/organ dysfunction [38]. Thus, a first DFD episode is heralding the severity of the cumulative unfavourable impact of the exposure to chronic hyperglycaemia in a given individual.

The results of our study should be considered with some limitations. Firstly, there were some missing values and the possibility of residual, unmeasured values due to the real-world primary healthcare nature of this data. Secondly, there is always a possibility of errors in the diagnostic registers due to the lack of standardization across health care professionals in the codification of health conditions and procedures related with the registry of the occurrence of DFD and registry of healing of DFD episode [14, 39]. However, robust statistical techniques were used, and we created well-balanced groups using propensity score matching as a valuable tool for addressing and enhancing the validity of causal inferences. Another limitation is the lack of specific CV mortality data, which could have impacted the estimation of the incident rates of mayor cardiovascular adverse events. For that reason, we created a composite outcome, including all-cause mortality. This strategy was previously used and shown to be viable [18, 40]. Moreover, there was limited information about socio-economic data, mental health conditions and no information on lifestyle variables for subjects, which could be valuable information. Additionally, data available from this cohort did not include codes for cancer events or cancer mortality.

## Conclusions

The results of this real-world study indicate that, independently from other risk factors, DFD is associated with higher morbidity and mortality. DFD is a severe complication of diabetes that heralds poor prognosis among people with T2DM. Future interventions should focus on preventing diabetes-related foot problems at early stage in order to improve the quality of life and survival.

### Electronic supplementary material

Below is the link to the electronic supplementary material.

Supplementary Material 1

Supplementary Material 2

## Data Availability

The data analysed in this study is subject to the following licenses/restrictions. Restrictions apply to the availability of some or all data generated or analysed during this study because they were used under license. Requests to access these datasets should be directed to Dr Dídac Mauricio, didacmauricio@gmail.com.

## References

[1] Lazzarini PA, Pacella RE, Armstrong DG, van Netten JJ (2018). Diabetes-related lower-extremity complications are a leading cause of the global burden of disability. Diabetes Med.

[2] Zhang Y, Lazzarini PA, McPhail SM, van Netten JJ, Armstrong DG, Pacella RE (2020). Global disability burdens of diabetes-related lower-extremity complications in 1990 and 2016. Diabetes Care.

[3] Armstrong DG, Swerdlow MA, Armstrong AA, Conte MS, Padula WV, Bus SA (2020). Five-year mortality and direct costs of care for people with diabetic foot complications are comparable to cancer. J Foot Ankle Res.

[4] McDermott K, Fang M, Boulton AJM, Selvin E, Hicks CW (2023). Etiology, epidemiology, and disparities in the burden of diabetic foot ulcers. Diabetes Care.

[5] Jeffcoate WJ, Vileikyte L, Boyko EJ, Armstrong DG, Boulton AJM (2018). Current challenges and opportunities in the prevention and management of diabetic foot ulcers. Diabetes Care.

[6] Rodríguez Pérez MDC, Chines C, Pedrero AJ, Cuevas FJ, Marcelino-Rodríguez I, Domínguez S, Cabrera De León A (2020). Major amputations in type 2 diabetes between 2001 and 2015 in Spain: Regional differences. BMC Public Health.

[7] Margolis DJ, Hoffstad O, Nafash J, Leonard CE, Freeman CP, Hennessy S, Wiebe DJ (2011). Location, location, location: Geographic clustering of lower-extremity amputation among Medicare beneficiaries with diabetes. Diabetes Care.

[8] Chamberlain RC, Fleetwood K, Wild SH, Colhoun HM, Lindsay RS, Petrie JR, McCrimmon RJ, Gibb F, Philip S, Sattar N, Kennon B, Leese GP (2022). Foot ulcer and risk of lower limb amputation or death in people with diabetes: a national population-based retrospective cohort study. Diabetes Care.

[9] Jeffcoate W, Barron E, Lomas J, Valabhji J, Young B (2017). Using data to tackle the burden of amputation in diabetes. Lancet.

[10] Røikjer J, Werkman NCC, Ejskjaer N, van den Bergh JPW, Vestergaard P, Schaper NC, Jensen MH, Klungel O, de Vries F, Nielen JTH, Driessen JHM (2022). Incidence, hospitalization, and mortality and their changes over time in people with a first-ever diabetic foot ulcer. Diabetes Med.

[11] Saluja S, Anderson SG, Hambleton I, Shoo H, Livingston M, Jude EB, Lunt M, Dunn G, Heald AH (2020). Foot ulceration and its association with mortality in diabetes mellitus: a meta-analysis. Diabet Med.

[12] Zhang P, Lu J, Jing Y, Tang S, Zhu D, Bi Y (2017). Global epidemiology of diabetic foot ulceration: a systematic review and meta-analysis. Ann Med.

[13] Ramsey SD, Newton K, Blough D, McCulloch DK, Sandhu N, Reiber GE, Wagner EH (1999). Incidence, outcomes, and cost of foot ulcers in patients with diabetes. Diabetes Care.

[14] Bundó M, Vlacho B, Llussà J, Puig-Treserra R, Mata-Cases M, Cos X, Jude EB, Franch-Nadal J, Mauricio D (2022). Prevalence and risk factors of diabetic foot disease among people with type 2 diabetes using real-world practice data from Catalonia during 2018. Front Endocrinol (Lausanne).

[15] Bolíbar B, Fina Avilés F, Morros R, Garcia-Gil Mdel M, Hermosilla E, Ramos R, Rosell M, Rodríguez J, Medina M, Calero S, Prieto-Alhambra D, Grupo SIDIAP (2012). [SIDIAP database: electronic clinical records in primary care as a source of information for epidemiologic research]. Med Clin (Barc).

[16] Mata-Cases M, Mauricio D, Real J, Bolíbar B, Franch-Nadal J (2016). ¿Se Registra Y Clasifica Correctamente La Diabetes mellitus en atención primaria? Estudio Poblacional en Cataluña, España. Endocrinol Y Nutr.

[17] ICD10data.com. About ICD10data.com. http://www.icd10data.com/About. Accessed July 1st 2023.

[18] Rastogi A, Sudhayakumar A, Schaper NC, Jude EB (2023). A paradigm shift for cardiovascular outcome evaluation in diabetes: major adverse cardiovascular events (MACE) to major adverse vascular events (MAVE). Diabetes Metab Syndr.

[19] Ho D, Imai K, King G, Stuart E, MatchIt. January: Nonparametric preprocessing for parametric causal inference. https://CRAN.R-project.org/package=MatchIt. Accessed 2022.

[20] Subirana I, Sanz H, Vila J. Building bivariate tables: The compareGroups package for R. J Stat Softw. 2014;57(12):1–16. https://www.jstatsoft.org/v57/i12/.

[21] Stevenson M, Nunes T, Heuer C, Marshall J, Sanchez J, Thornton R, Reiczigel J, Robison-Cox J, Sebastiani P, Solymos P. January. epiR: Tools for the analysis of epidemiological data. https://fvas.unimelb.edu.au/research/groups/veterinary-epidemiology-melbourne. Accessed 2022.

[22] R Core Team. R: A language and environment for statistical computing. Vienna, Austria: R Foundation for Statistical Computing. https://www.R-project.org/. Accessed January 2022.

[23] Armstrong DG, Boulton AJM, Bus SA (2017). Diabetic foot ulcers and their recurrence. N Engl J Med.

[24] Dubský M, Jirkovská A, Bem R, Fejfarová V, Skibová J, Schaper NC, Lipsky BA (2013). Risk factors for recurrence of diabetic foot ulcers: prospective follow-up analysis in the Eurodiale subgroup. Int Wound J.

[25] Boyko EJ, Seelig AD, Ahroni JH (2018). Limb- and person-level risk factors for lower-limb amputation in the prospective Seattle Diabetic Foot Study. Diabetes Care.

[26] Pecoraro RE, Reiber GE, Burgess EM (1990). Pathways to diabetic limb amputation. Basis for prevention. Diabetes Care.

[27] Jeffcoate WJ, Harding KG (2003). Diabetic foot ulcers. Lancet.

[28] Margolis D, Malay DS, Hoffstad OJ, Leonard CE, MaCurdy T, Lopez de Nava K, Tan Y, Molina T, Siegel KL. January. Incidence of diabetic foot ulcer and lower extremity amputation among Medicare beneficiaries, 2006 to 2008. Data Points #2 (prepared by the University of Pennsylvania DEcIDE Center, under Contract No. HHSA29020050041I). Rockville, MD: Agency for Healthcare Research and Quality. 2011. AHRQ Publication No. 10(11)-EHC009-1-EF.

[29] Chuter V, Schaper N, Mills J, Hinchliffe R, Russell D, Azuma N, Behrendt CA, Boyko EJ, Conte MS, Humphries M, Kirksey L, McGinigle KC, Nikol S, Nordanstig J, Rowe V, van den Berg JC, Venermo M, Fitridge R (2023). Effectiveness of bedside investigations to diagnose peripheral artery disease among people with diabetes mellitus: a systematic review. Diabetes Metab Res Rev.

[30] Bundó M, Llussà J, Serra M, la Iglesia PP, Gimbert RM, Real J, Vlacho B, Mata-Cases M, Cos X, Franch-Nadal J, Mauricio D (2021). Incidence and characteristics of diabetic foot ulcers in subjects with type 2 diabetes in Catalonian primary care centres: an observational multicentre study. Prim Care Diabetes.

[31] Chen L, Sun S, Gao Y, Ran X (2023). Global mortality of diabetic foot ulcer: a systematic review and meta-analysis of observational studies. Diabetes Obes Metab.

[32] Boyko EJ, Ahroni JH, Smith DG, Davignon D (1996). Increased mortality associated with diabetic foot ulcer. Diabet Med.

[33] Walsh JW, Hoffstad OJ, Sullivan MO, Margolis DJ (2016). Association of diabetic foot ulcer and death in a population-based cohort from the United Kingdom. Diabet Med.

[34] Muller IS, De Grauw WJC, Van Gerwen WHEM, Bartelink ML, van Den Hoogen HJ, Rutten GE (2002). Foot ulceration and lower limb amputation in type 2 diabetic patients in Dutch primary health care. Diabetes Care.

[35] Winkley K, Stahl D, Chalder T, Edmonds ME, Ismail K (2007). Risk factors associated with adverse outcomes in a population-based prospective cohort study of people with their first diabetic foot ulcer. J Diabetes Complications.

[36] Alonso-Moran E, Orueta JF, Fraile Esteban JI (2014). The prevalence of diabetes-related complications and multimorbidity in the population with type 2 diabetes mellitus in the Basque Country. BMC Public Health.

[37] Mauricio D, Gratacòs M, Franch-Nadal J (2023). Diabetic microvascular disease in non-classical beds: the hidden impact beyond the retina, the kidney, and the peripheral nerves. Cardiovasc Diabetol.

[38] Mauricio D, Alonso N, Gratacòs M (2020). Chronic diabetes complications: the need to move beyond classical concepts. Trends Endocrinol Metab..

[39] Hoffstad O, Mitra N, Walsh J, Margolis DJ (2015). Diabetes, lower-extremity amputation, and death. Diabetes Care.

[40] Real J, Vlacho B, Ortega E (2021). Cardiovascular and mortality benefits of sodium-glucose co-transporter-2 inhibitors in patients with type 2 diabetes mellitus: CVD-Real Catalonia. Cardiovasc Diabetol.

