# Supplementary material for: Diabetic foot disease carries an intrinsic high risk of mortality and other severe outcomes in type 2 diabetes: a propensity score-matched retrospective population-based study
Source: Cardiovasc Diabetol. 2024 Jun 19;23:209. doi: 10.1186/s12933-024-02303-1 (PMC11188162; doi:10.1186/s12933-024-02303-1)
Supplement: Supplementary file 2 — Supplementary Material 2 [file 12933_2024_2303_MOESM2_ESM.docx]

**SUPPLEMENTARY MATERIAL**

Diabetic Foot Disease carries an intrinsic high risk of mortality and other severe outcomes in Type 2 Diabetes: A Propensity Score-Matched Retrospective Population-based Study

**Bogdan Vlacho, Magdalena Bundó, Judit Llussà, Jordi Real, Manel Mata-Cases, Xavier Cos, Diana Tundidor, Francesco Zaccardi, Edward B. Jude, Josep Franch-Nadal, Dídac Mauricio**

| **Title** | **Page** |
| --- | --- |
| *Supplementary Table 1.1* Definition of DFD | 2 |
| *Supplementary Table 1.2* Definition of events | 4 |
| *Supplementary Figure 1* Flowchart | 6 |
| *Supplementary Figure 2.*1 Propensity score matching | 7 |
| *Supplementary Figure 2.2* Density plot for Propensity score matching | 8 |
| *Supplement Table 2.* Unadjusted and adjusted hazard ratios for different study events | 9 |
| *Supplementary table 3.* Sub-analysis of outcomes regarding the severity of the DFD | 10 |

Supplementary Table 1.1 Definition for DFD

| Diabetic foot disease | Variable | Codes |
| --- | --- | --- |
|  | Lower-extremity ulcers | L97529; L97524; L97523; L97522; L97521; L9752; L97519; L97514; L97513; L97512; L97511; L9751; L97509; L97504; L97503; L97502; L97501; L9750; L97422; L97421; L9742; L97419  L97414; L97413; L97412; L97411  L9741; L97409; L97404; L97403; L97402; L97401; L9740; L974; L97; 70719; 70715; 70714; 70710; 7071; L97; E11.621 |
|  | Osteomyelitis | M868X6;M868X7; M86679;M86672; M86671;M8667; M86479; M86472; M86471; M8647; M8640; M86379; M86372; M86371; M8637; M86272; M86279; M86271; M8627; M86179; M86172; M86171; M8617; 73097; 73087; 73037; 73027; 73026; 73017; 73016; 73007; 73006; M86 |
|  | Gangrene | I96; E1152; 7854; R02 |
|  | Lower-extremity amputation and surgical detachment procedures related to lower-extremity amputations | S98.4; Y83.5; Z89.4; Z89.41  Z89.411; Z89.412; Z89,419; Z89.42; Z89.421; Z89.422; Z89,429; Z89.43; Z89.431  Z89.432; Z89,439; Z89.44  Z89.441; Z89,442; Z89,449; Z89.5; Z89.51; Z89.511;; Z89.512; Z89,519; Z89.6  Z89.61; Z89.611; Z89.612; Z89,619; Z89.62; Z89.621;; Z89.622; Z89,629; 841; 8410; 8411; 8412; 8414; 8415; 8;  8491;Y670ZZ; 0Y680ZZ;Y6C0Z1; 0Y6C0Z2; 0Y6C0Z3;0Y6D0Z1; 0Y6D0Z2; 0Y6D0Z3; 0Y6F0ZZ; 0Y6G0ZZ;; 0Y6H0Z1; 0Y6H0Z2  0Y6H0Z3; 0Y6J0Z1; 0Y6J0Z2; 0Y6J0Z3;0Y6M0Z0; 0Y6M0Z4;0Y6M0Z5; 0Y6M0Z6; 0Y6M0Z7;0YM0Z8; 0Y6M0Z9; 0Y6M0ZB;0Y6M0ZC;0Y6M0ZD; ;0Y6M0ZF; 0Y6N0Z0; 0Y6N0Z4; 0Y6N0Z5; 0Y6N0Z6; 0Y6N0Z7; 0Y6N0Z8; 0Y6N0Z9; 0Y6N0ZB; 0Y6N0ZC; 0Y6N0ZD; 0Y6N0ZF;0Y6P0Z0; 0Y6P0Z1; 0Y6P0Z2; 0Y6P0Z3; 0Y6Q0Z0; 0Y6Q0Z1; Y6Q0Z2; 0Y6Q0Z3; 0Y6R0Z0; 0Y6R0Z1; 0Y6R0Z2; 0Y6R0Z3; 0Y6S0Z0; 0Y6S0Z1; 0Y6S0Z2; 0Y6S0Z3; 0Y6T0Z0; 0Y6T0Z1; 0Y6T0Z2; 0Y6T0Z3; 0Y6U0Z0; 0Y6U0Z1; 0Y6U0Z2  0Y6U0Z3; 0Y6V0Z0; 0Y6V0Z1; 0Y6V0Z2; 0Y6V0Z3; 0Y6W0Z0; 0Y6W0Z1; 0Y6W0Z2; 0Y6W0Z3; 0Y6X0Z0; 0Y6X0Z1  0Y6X0Z2; 0Y6X0Z3; 0Y6Y0Z0; 0Y6Y0Z1; 0Y6Y0Z2; 0Y6Y0Z3; Y83.5; Z89.4; Z89.5 |
|  | Charcot neuroarthropathy | M1469; M14679; M14672; M14671; M1467; M1460; M146; 7135; M14.6 |

Table 1.2 Definition of events

| Clinically relevant events | new episodes of DFD | Lower-extremity ulcers and/or  Osteomyelitis and/or  Gangrene and/or  Lower-extremity amputation and/or surgical detachment procedures related to lower-extremity amputations and/or Charcot neuroarthropathy during the follow-up |
| --- | --- | --- |
|  | cardiovascular events (stroke, ischemic heart disease, peripheral arteriopathy, heart failure), | I798; I79; I739; I7389; I738; I73; 44389; 44381; I70.2; I70.20; I70.201; I70.202; I70.203; I70.208; I70.209; I70.21; I70.211; I70.212; I70.213; I70.218; I70.219  I70.22; I70.221; I70.222; I70.223; I70.228; I70.229; I70.23;70.231; I70.232; I70.233; I70.234; I70.235; I70.238; I70.239; I70.24; I70.241; I70.242; I70.243; I70.244; I70.245; I70.248; I70.249; I70.261; I70.262; I70.263; I70.291; I70.292; I70.293; I72.4; I79.8; I79.2; I79; I74.5; I74.3; I73.9; I70.2; I69.8; I69.4; I69.3; I69.1; I67.8; I63.9; I63.5; I63.2; I63; I62.9; I62; I61.8; I61.5; I61.4; I61.3; I61.1; I61.0; I61; I60.8; I60.7; I60.5; I60.4; I60.3; I60.1; I50.91; I25.9; I25.1; I25; I24.9; I24.0; I24; I23.6; I23.1  I23.0; I23; I22.9; I22.8; I21.4; I20; I13.2; I13.0; G46.8; G46.7; G46.3  G46.2; G46.0; G45.3; G45.0; I74.4; I73.9; I73.8; I69.2; I69.0; I69; I67.9; I64; I63.8; I63.6; I63.4; I63.3; I63.1; I63.0; I61.9; I61.6  I61.2; I60.9; I60.6; I60.2; I60.0; I60; I50.90; I50.9; I50.1; I50.0; I50; I25.8; I25.6; I25.5; I25.4; I25.3; I25.2; I25.0; I24.8; I23.8  I23.5; I23.4; I23.3; I23.2; I22.1  I22.0; I22; I21.9; I21.3; I21.2  I21.1; I21.0; I21; I20.9; I20.8; I20.0; I11.0; G46.6; G46.5; G46.4  G46.1; G46; G45.9; G45.8; G45.4; G45.2; G45.1; T82.2 |
|  | amputations | Lower-extremity amputation and/or surgical detachment procedures related to lower-extremity amputations during the follow-up |
|  | all-cause mortality | Mortality register |
|  | Composite outcome | Mortality and/or cardiovascular events |

Supplement Figure 1. Flowchart of cohort definition


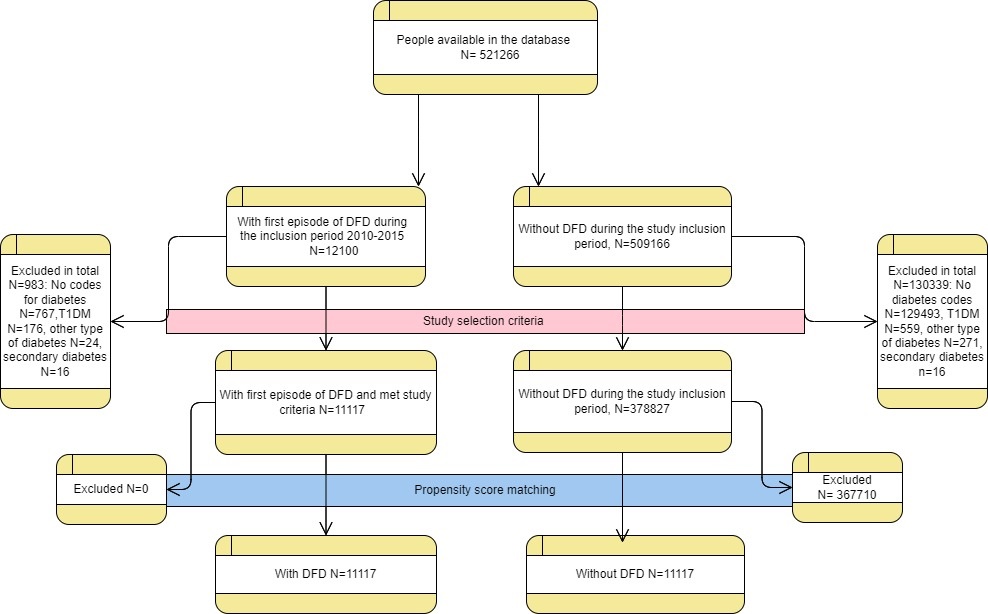


Study selection criteria: all subjects from the database with a diagnosis of T2DM during the inclusion period. T2DM was defined by the presence of ICD-10 diagnostic codes (International Statistical Classification of Diseases and Related Health Problems, 10th Revision) E11 and E14. Subjects with other types of diabetes (ICD-10: type 1 diabetes, E10; malnutrition-related diabetes, E12; gestational diabetes, O24; other specific types of diabetes, E13) were excluded from the analysis.

Supplement Figure 2.1 Standardised differences of variable included in the propensity score


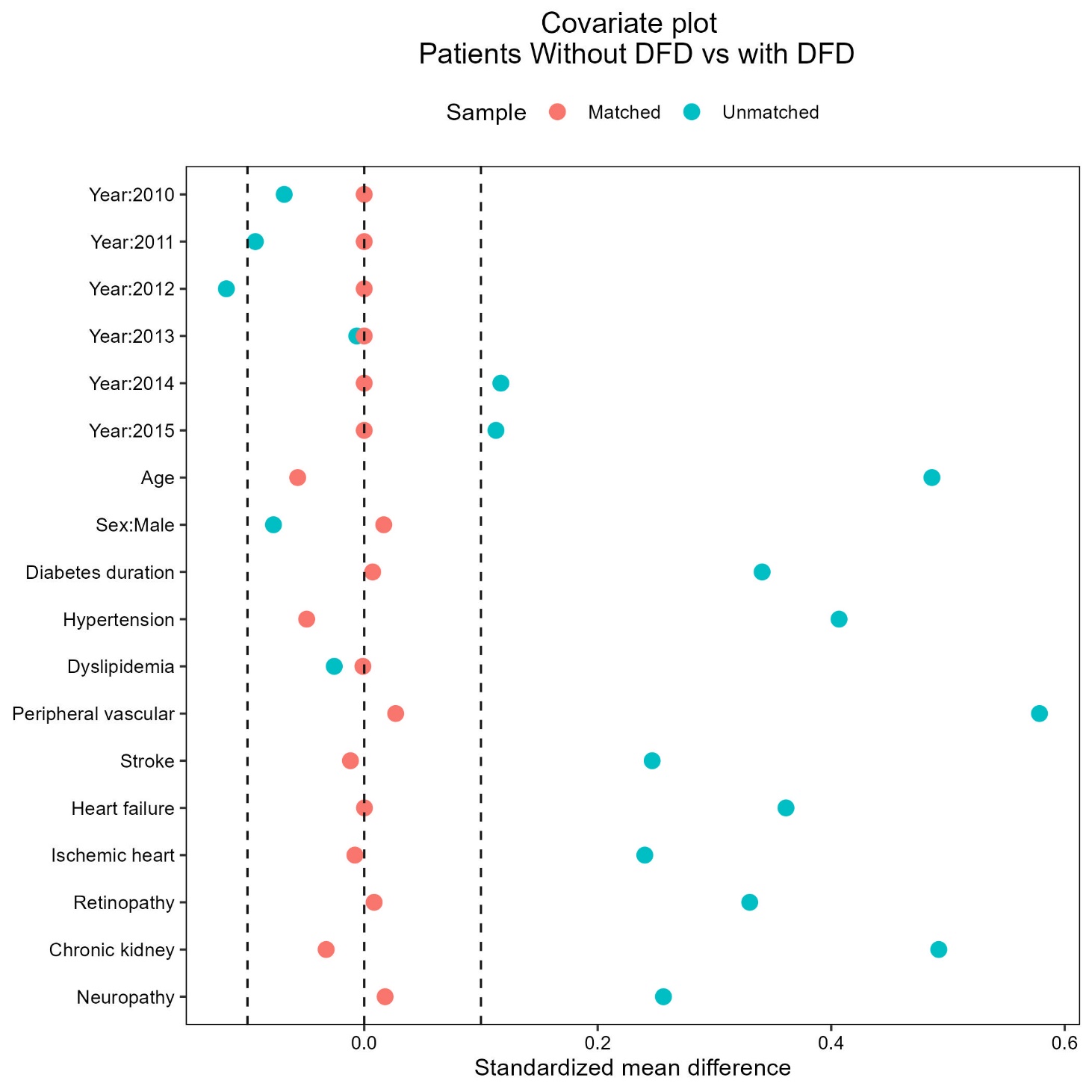


Supplement Figure 2.2. Density plot before and after matching


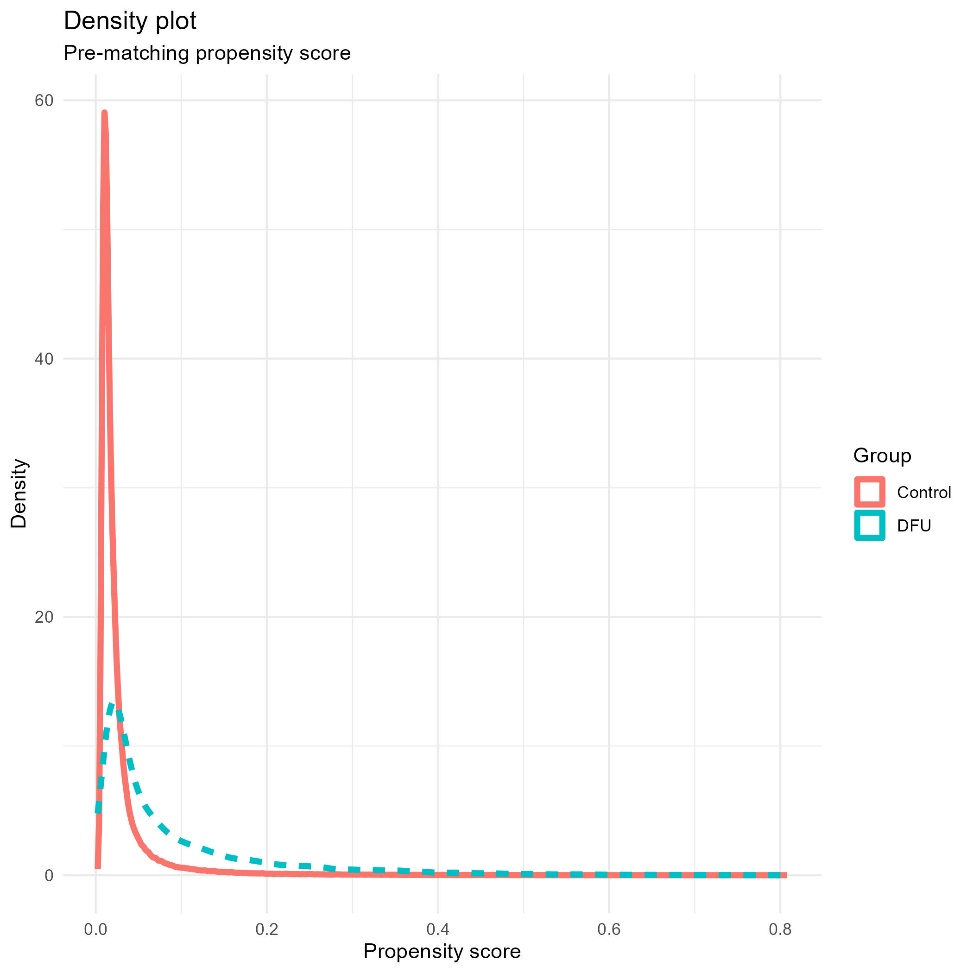

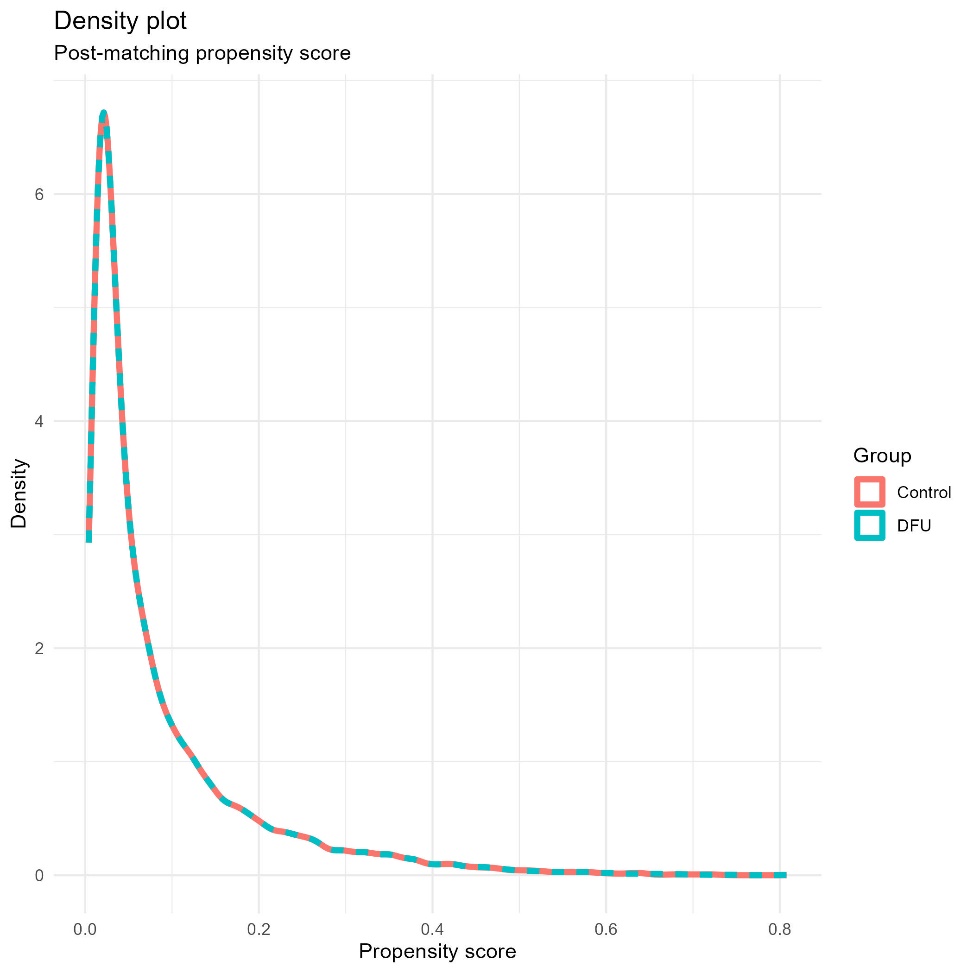


**Supplement Table 2.** Unadjusted and adjusted hazard ratios for different study events

| **Event** | **Group DFD** | **HR** | **Lower CI** | **Upper CI** |
| --- | --- | --- | --- | --- |
| New DFD episode | Unadjusted | 15.14 | 13.85 | 16.56 |
|  | Adjusted | 15.03 | 13.74 | 16.44 |
| Amputations | Unadjusted | 18.74 | 16.22 | 21.64 |
|  | Adjusted | 17.82 | 15.42 | 20.59 |
| All-cause mortality | Unadjusted | 1.97 | 1.89 | 2.05 |
|  | Adjusted | 2.24 | 2.15 | 2.33 |
| Cardiovascular disease | Unadjusted | 2.25 | 2.15 | 2.36 |
|  | Adjusted | 2.27 | 2.17 | 2.38 |
| Composite outcome | Unadjusted | 2.20 | 2.12 | 2.27 |
|  | Adjusted | 2.30 | 2.22 | 2.38 |

Adjusted for: Age at index date, sex, smoking status, alcohol consumption, diabetes duration, hypertension, hyperlipidemia, chronic kidney disease, microvascular complications, BMI, HbA1c. *ref= With DFD (diabetic foot disease); composite outcome: all-cause mortality and/or cardiovascular disease

**Supplementary table 3.** Sub-analysis of outcomes regarding the severity of the DFD

| Event | New DFD episode | | Amputations | | All-cause mortality | | Cardiovascular disease | | Composite outcome | |
| --- | --- | --- | --- | --- | --- | --- | --- | --- | --- | --- |
| Baseline condition | With foot ulcers | With other DFD | With foot ulcers | With other DFD | With foot ulcers | With other DFD | With foot ulcers | With other DFD | With foot ulcers | With other DFD |
| Number of events | 3039 | 1867 | 1220 | 1449 | 4312 | 1801 | 2967 | 1528 | 5795 | 2569 |
| Person years | 15925.21 | 6126.08 | 20280.57 | 7441.80 | 23894.54 | 12446.20 | 16562.05 | 8087.66 | 16562.05 | 8087.66 |
| IR  [95% CI] | 19.08 [18.41;19.77] | 30.48  [29.11;  31.89] | 6.02  [5.68;  6.36] | 19.47  [18.48;  20.50] | 18.05  [17.51;  18.59] | 14.47  [13.81;  15.15] | 17.91  [17.28;  18.57] | 18.89  [17.96;  19.86] | 34.99  [34.09;  35.90] | 31.76  [30.55;  33.02] |
| IRR  [95% CI] | 1.60 [1.51;1.60] | | 3.24 [3.00;3.24] | | 0.8 [0.76; 0.80] | | 1.05 [0.99; 1.05] | | 0.91 [0.87; 0.91] | |
| HR*  [95% CI] | 1.43 [1.35; 1.52] | | 2.66 [2.46; 2.88] | | 1.02 [0.96; 1.08] | | 1.05 [0.99;1.13] | | 1.02 [0.97;1.07] | |

DFD: diabetic foot disease; composite outcome: all-cause mortality and/or cardiovascular disease; the group of subjects with other DFD in this sub-analysis included subjects with any of the following conditions: osteomyelitis, gangrene, Charcot neuroarthropathy or lower-extremity amputation)* HR Adjusted for: Age at index date, sex, smoking status, alcohol consumption, diabetes duration, hypertension, hyperlipidaemia, chronic kidney disease, microvascular complications, BMI, HbA1c. ref= With foot ulcers
